# Effectiveness of nationwide screening and lifestyle intervention for abdominal obesity and cardiometabolic risks in Japan: The metabolic syndrome and comprehensive lifestyle intervention study on nationwide database in Japan (MetS ACTION-J study)

**DOI:** 10.1371/journal.pone.0190862

**Published:** 2018-01-09

**Authors:** Yoko M. Nakao, Yoshihiro Miyamoto, Kenji Ueshima, Kazuhiro Nakao, Michikazu Nakai, Kunihiro Nishimura, Shinji Yasuno, Kiminori Hosoda, Yoshihiro Ogawa, Hiroshi Itoh, Hisao Ogawa, Kenji Kangawa, Kazuwa Nakao

**Affiliations:** 1 Department of Preventive Medicine and Epidemiologic Informatics, National Cerebral and Cardiovascular Center, Suita, Osaka, Japan; 2 Institute for Advancement of Clinical and Translational Science, Kyoto University Hospital, Kyoto, Kyoto, Japan; 3 Department of Cardiovascular Medicine, National Cerebral and Cardiovascular Center, Suita, Osaka, Japan; 4 Department of Statistics and Data Analysis, Center for Cerebral and Cardiovascular Disease Information, National Cerebral and Cardiovascular Center, Suita, Osaka, Japan; 5 Department of Endocrinology and Metabolism, National Cerebral and Cardiovascular Center, Suita, Osaka, Japan; 6 Department of Molecular Endocrinology and Metabolism, Graduate School of Medical and Dental Sciences, Tokyo Medical and Dental University, Tokyo, Japan; 7 Department of Internal Medicine, Keio University School of Medicine, Tokyo, Japan; 8 National Cerebral and Cardiovascular Center, Suita, Osaka, Japan; 9 Medical Innovation Center, Kyoto University School of Medicine, Kyoto, Kyoto, Japan; University College London, UNITED KINGDOM

## Abstract

**Background:**

Lifestyle interventions can substantially improve obesity and cardiometabolic risks. However, evidence of long-term benefits of national intervention is sparse. We aimed to evaluate the long-term effectiveness of a nationwide program for abdominal obesity.

**Methods:**

A retrospective cohort study was performed using a longitudinal nationwide individual data in subjects aged 40–74 years who underwent checkups in fiscal year (FY) 2008. Lifestyle interventions were provided via interview in subjects with abdominal obesity and at least one cardiometabolic risk factor. Subjects who attended the lifestyle intervention (participants) were compared to those who did not attend (non-participants). Outcomes were waist circumferences (WC) and body mass index (BMI) reduction, reversal of metabolic syndrome (MetS), and changes in cardiometabolic risks. We used a three-step process with robust analytic approaches to account for selection bias that included traditional multivariate analysis, propensity-score matching and instrumental variable (IV) analyses.

**Results:**

Of 19,969,722 subjects, 4,370,042 were eligible for analyses; 111,779 participants and 907,909 non-participants. A higher percentage of participants had ≥5% reductions in obesity profiles at year 3, compared to non-participants (WC, 21.4% vs 16.1%; BMI, 17.6% vs 13.6%; p<0.001 each). Participants also had higher reversal for MetS (adjusted odds ratio 1.31; 95% confidence interval: 1.29–1.33; p<0.001). Greater reductions in cardiometabolic risks were observed in participants. Those results were confirmed in analyses using a propensity score-matched cohort (n = 75,777, each) and IV analyses. Limitations of this work include the use of non-randomized national data in Japan to assess the effectiveness of the nationwide preventive program.

**Conclusions:**

In the nationwide lifestyle intervention for abdominal obesity, the at-risk population achieved significant reductions in WC, BMI, and cardiometabolic risks in 3 years. This study provides evidence that the nationwide program effectively achieved long-term improvement in abdominal obesity and cardiometabolic risks.

## Introduction

The worldwide incidence and economic effect of cardiovascular disease (CVD) are substantial. Healthcare providers must focus on reducing CVD risk factors by helping individuals begin and maintain lifestyle changes [1].

Published data strongly support the benefits of lifestyle changes as a means to prevent CVD [1, 2]. Interventions that target lifestyle changes often result in impressive rates of initial behavior changes. However, population changes in lifestyle can be difficult to achieve in clinical practice [2]. Particularly, behavioral lifestyle changes are frequently not translated into long-term behavioral changes [3]. Both adoption and maintenance of new cardiometabolic risk-reducing behaviors pose challenges for many individuals [1]. Therefore, nationwide changes in healthcare policies are needed to translate the evidence into action and improve CVD prevention at personal, organizational, social, and political levels in many sectors [4–6].

Since April 2008, Japan has embarked on a national health policy change to prevent lifestyle-related diseases, such as CVD and diabetes [7]. This comprehensive preventive policy involves an unprecedented nationwide screening and lifestyle intervention for abdominal obesity with more than 100 million people in a developed country. Health checkups, which focused on abdominal obesity and cardiometabolic risk factors, were performed annually for individuals, or their family members, aged 40–74 years and covered as primary beneficiaries under the health insurance system. From April 2008 to March 2012, 45,313,284 individuals had specific health checkups, accounting for approximately 86% of the Japanese population of comparable age.

Here, we evaluated the long-term effectiveness of this nationwide program to prevent abdominal obesity and CVD risks in a community-based setting. Assessment of the potential effectiveness of lifestyle intervention requires caution because of selection bias, which could arise when highly health-conscious or motivated subjects are selected to receive lifestyle interventions. We therefore used a three-step process with robust analytic approaches to account for bias that included traditional multivariate analysis, propensity-score matching, and instrumental variable (IV) analysis with lifestyle intervention participation rates as the instrument.

## Materials and methods

### Study design and population

We conducted a nationwide cohort study, known as the Metabolic Syndrome And Comprehensive lifesTyle Intervention study On Nationwide database in Japan (MetS ACTION-J) with retrospectively collected data from the National Database of Health Insurance Claims and Specific Health Checkups of Japan (NDB). The national data captured examination records and laboratory data to diagnose MetS. We obtained anonymized data regarding subjects who underwent a screening program between fiscal year 2008 and fiscal year 2011 from the Ministry of Health, Labour and Welfare (MHLW). We have created a database for this study (**S1 Appendix**). The ethics committees of the University of Kyoto School of Medicine and National Cerebral and Cardiovascular Center approved the study. Written informed consent was not obtained due to the retrospective design.

We considered all subjects aged 40–74 years who completed the screening visits in fiscal year 2008. We excluded subjects who were receiving medications for hypertension or dyslipidaemia or diabetes, those who meet the diabetes criteria (fasting blood glucose [FBG] ≥126 and/or hemoglobin A1c [HbA1c] ≥6.5%), those without data required for diagnosing metabolic syndrome (MetS), and those who did not have the specific health checkup in fiscal year 2011.

### Screening and lifestyle interventions

All subjects received health checkups, including questionnaires, physical examinations, and measurements of height and weight, waist circumference (WC), blood pressure, serum cholesterol, blood glucose, and/or HbA1c. All exams were conducted under an article prescribed by an ordinance of the MHLW (**S2** and **S3 Appendices**). Smoking status was determined by self-reported questionnaires. Exams, including anthropometry measurements, blood pressure, and blood samples, were carried out after at least a 10-hour fast, and the subjects were instructed to perform no physical activities of moderate or high intensity and to ingest no caffeine and alcohol the day before the test. WC was measured at the umbilical level in a standing position using a tape measure after normal expiration (**S2 Appendix**). If the umbilical level was displaced downward due to accumulation of abdominal fat, WC was measured at the midpoint between the superior border of the iliac crest and the inferior margin of the twelfth rib. Blood pressure was measured twice after at least 5 min rest with the participant seated. Regardless of the presence/absence of risk, information and advice on CVD prevention were given to all subjects simultaneously with notification of the checkup results annually or more frequently. Specific health promotion guidance was provided to at-risk individuals. Individuals at risk were defined as those with a WC ≥85 cm for men and ≥90 cm for women [8] and/or body mass index (BMI) ≥25 kg/m^2^ who satisfy at least one of the following requirements: (1) elevated blood pressure (systolic blood pressure [SBP] ≥130 mmHg or diastolic blood pressure [DBP] ≥85 mmHg), (2) dyslipidaemia (triglycerides [TG] ≥150 mg/dl or high-density lipoprotein [HDL]-cholesterol <40 mg/dl), and/or (3) impaired glucose tolerance (FBG ≥100 mg/dl or HbA1c ≥5.6%). After subjects with risks received health checkup results by mail or other means, they reserved counselling sessions themselves. Health promotion guidance was provided via interview by healthcare providers (physician, health nurse, or managerial dietician) with or without additional continuous support for 3 months or longer in response to individual risks (**Table 1**and **S3 Appendix**).

**Table 1:** Nationwide preventive program: Screening and lifestyle intervention features.

**Health checkups**
**Subjects** - All individuals or their family members aged 40–74 years, who are covered as primary beneficiaries under the health insurance system in Japan. - Pregnant women, prisoners, individuals living overseas, and long-term inpatients were excluded. **Exams** - Exams included questionnaires, physical examinations, waist circumference, height, weight, blood pressure, blood samples, and urinalyses. - All exams were conducted under an article prescribed by an ordinance of the Ministry of Health, Labour and Welfare. **Results & Information Supply** - Regardless of the presence/absence of cardiometabolic risks, information was given to all subjects simultaneously, with notification of the checkup results annually or more frequently.
**Lifestyle interventions**
- Individuals with a waist circumference ≥85 cm (men)/≥90 cm (women) or <85 cm (men)/<90 cm (women) with a body mass index ≥25 kg/m^2^, with at least one of the following: (1) high glucose tolerance (fasting blood glucose ≥100 mg/dl or hemoglobin A1c ≥5.6%), (2) dyslipidemia (triglyceride ≥150 mg/dl or high-density lipoprotein-cholesterol <40 mg/dl), (3) high blood pressure (systolic blood pressure ≥130 mm Hg or diastolic blood pressure ≥85 mm Hg) were considered at risk. - Individuals taking medications for hypertension, dyslipidemia, or diabetes mellitus were excluded. **Interventions** - Interventions were provided for 20 minutes or more to each individual separately or for 80 minutes or more to a group by a physician, public health nurse, or registered dietician. - The facilitator explained the necessity of lifestyle improvement, the relationship between lifestyle and the specific health checkup data, the patient’s lifestyle, knowledge about metabolic syndrome and lifestyle-related chronic diseases, and the influence of these factors on the daily lives of the individuals receiving the motivational support. - Explanation was given about the advantages of lifestyle improvement and the disadvantages of failing to improve lifestyle. - The facilitator suggested changes needed to improve the lifestyle (e.g., diet and exercise). - The facilitator set goals for actions and the timing of the outcome evaluation, accompanied by presentation of the social resources needed for lifestyle improvement and support for their effective utilization. - The facilitator showed how to measure body weight and abdominal circumference. - Goals for actions and the action plan were prepared by the individual receiving the motivational support under guidance via interview. - A follow-up interview was performed based on the risks. - The evaluation was made via interview or telecommunication (telephone, e-mail, etc.), 6 months after the first session.

### Statistical analysis

We evaluated WC and BMI reduction, and reversal of MetS using logistic regression models and changes in cardiometabolic risk factors using linear regression models to investigate associations between lifestyle intervention and cardiometabolic risk factors. Since previous studies have shown that a loss of 5–10% of initial weight is associated with significant reductions in cardiovascular risks, a 5% reduction was considered clinically meaningful [9]. Reversal of MetS was defined as normalization for subjects with MetS [8] and risk reduction for subjects with pre-MetS. We performed regression analysis with coefficients expressed per one standard deviation (SD) to compare intervention effect size directly among cardiometabolic risk factors. Covariates included age, sex, and smoking status as categorical variables, and BMI (except the WC model) and baseline individual component level as continuous variables. In addition to adjusting for covariates, we performed rigorous adjustment for baseline variables using propensity-score matching. The propensity score was calculated from a multivariate probit regression model in the whole cohort that included demographic characteristics and cardiometabolic risk factors. Matching was performed using a 1:1 matching protocol within a calliper of 0.01 SD of the propensity score probit.

We performed IV analysis to provide estimates that would remain unbiased even if important confounding variables were not measured [10]. We used the facility level ratio of lifestyle intervention, in which the facility intervention ratio was defined as the number of intervention participants divided by the total number of candidates, as the instrument. Similar instruments have previously been reported [11]. In this study, it is unlikely that the facility ratios of lifestyle intervention would be associated with clinical improvements in any way other than the participants of intervention. To confirm that the ratio of lifestyle intervention was not a weak instrument, we used a partial F test and found sufficiently large F statistics. Patients seen at institutions with an intervention rate less than 0.1% and over 60% were excluded from the IV analysis, since lifestyle intervention choice may have been decided less by facility preference and therefore be subject to confounding by unmeasured subject-level covariates [12]. We evaluated the balance of measured covariates across levels of the IV to provide additional information to assess its validity (**S2 Table**). Using two-stage linear regression for the calculation of the coefficients, we estimated IV-adjusted changes of cardiometabolic factors with the IV being the facility intervention rate, using the STATA procedure IVREG. Covariates include geographic location and clinical characteristics, including urban or rural (**S4 Appendix**), age, sex, smoking status, BMI (except the WC), and baseline individual component level. The analyses were conducted using STATA, 13.1 (STATA Corp., College Station, Texas) and SAS 9.2 (SAS Institute, Cary, North Carolina).

## Results

### Study population

**Fig 1:**
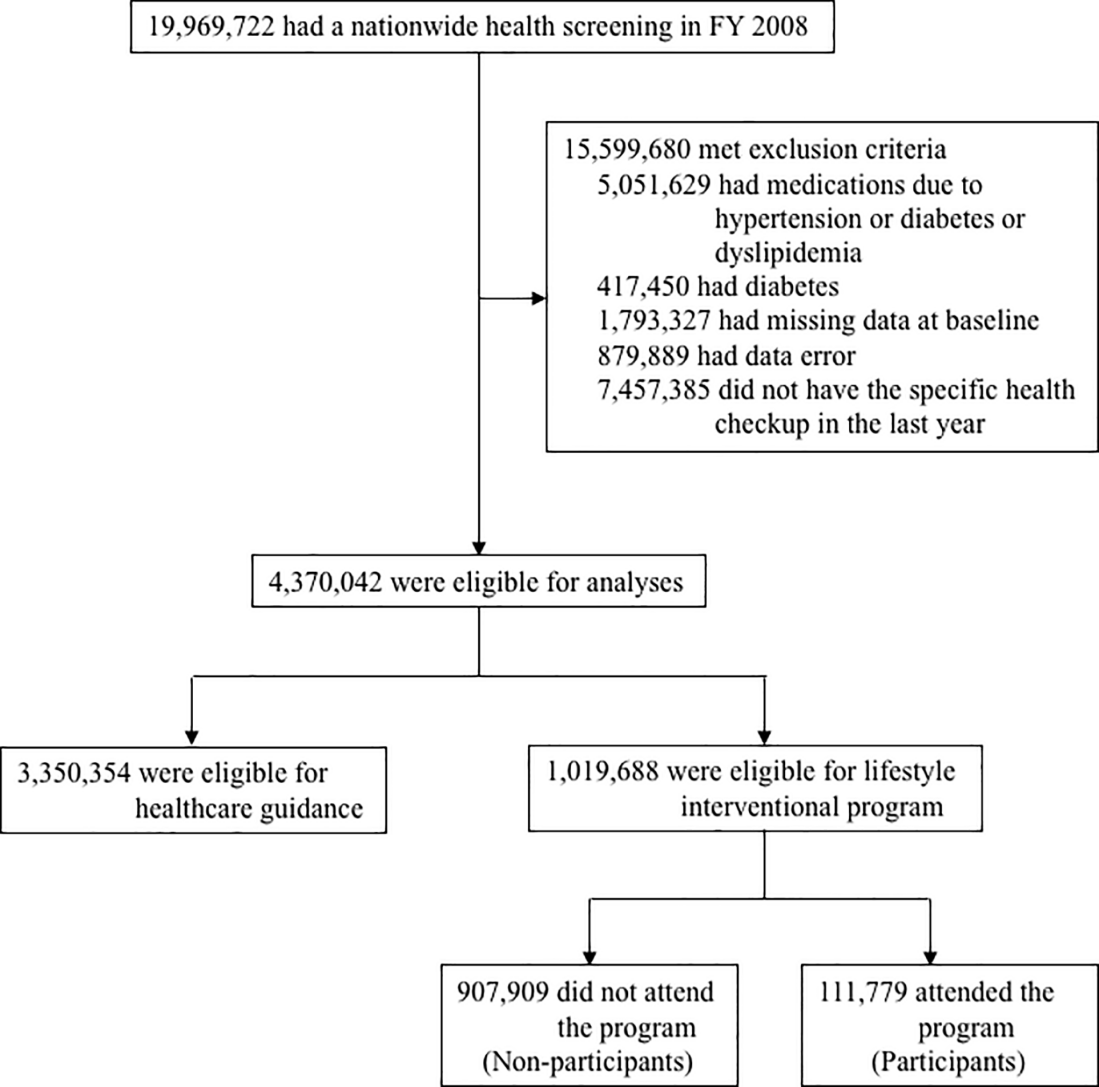
Study flow chart. FY, fiscal year.

We included subjects (n = 19,969,722) aged 40–74 years who completed the screening visits in the first year (from April 2008 to March 2009). Among this population, we excluded 15,599,680 subjects who were receiving medications for hypertension or dyslipidemia or diabetes (n = 5,051,629), those who meet diabetes criteria (n = 417,450), those without appropriate data required for diagnosing MetS (n = 1,793,327), those with data error (n = 879,889), and those who did not have the specific health checkup in fiscal year 2011 (n = 7,457,385). Of 4,370,042 subjects who were eligible for analyses, 3,350,354 (76.7%) were eligible for healthcare guidance (information supply only), and 1,019,688 (23.3%) were eligible for the interventional program. Totally, 111,779 subjects attended the health guidance program (participants), and 907,909 did not (non-participants) (**Fig 1**).

At baseline, the mean (SD) WC and BMI were 91.2 (5.9) cm and 26.2 (2.5) kg/m^2^ for participants and 91.2 (5.7) cm and 26.1 (2.4) kg/m^2^ for non-participants, respectively. Non-participants had slightly worse blood pressures and lipid profiles than participants (**Table 2**). After propensity-score matching was performed for the entire population, there were 75,777 matched pairs. The absolute standardized differences were <10% for all variables entered into the propensity score, indicating adequate matches [13].

**Table 2:** Baseline characteristics.

Characteristics	No-risk group (eligible for healthcare guidance)	At-risk group (eligible for interventional program)			
		Non-participants	Participants	Standardized difference ^a^	P value (Non-participants vs. participants)
n	3 350 354	907 909	111 779		
Age, %					
40–44	27.4	24.9	18.9	−14.4	<0.001
45–49	23.3	24.1	19.8	−10.4	
50–54	18.9	21.7	18.0	−9.2	
55–59	12.2	13.9	13.2	−2.1	
60–64	7.6	6.5	10.2	13.4	
65–69	8.1	6.6	14.6	26.0	
≥70	2.5	2.3	5.3	15.8	
Men, %	51.6	82.4	77.8	11.5	<0.001
Smoking, %	25.0	34.9	28.4	14.0	<0.001
WC, cm	78.1 (7.1)	91.2 (5.9)	91.2 (5.7)	−1.0	0.001
BMI, kg/m^2^	21.6 (2.4)	26.2 (2.5)	26.1 (2.4)	−5.9	<0.001
SBP, mm Hg	118.2 (15.6)	130.7 (15.9)	130.6 (15.4)	−0.5	0.154
DBP, mm Hg	73.1 (10.7)	82.2 (11.0)	81.2 (10.5)	−9.9	<0.001
TG, mg/dl ^b^	82 (60–116)	147 (99–201)	141 (96–195)	−5.1	<0.001
HDL, mg/dl	66.3 (16.0)	54.0 (13.2)	54.1 (13.1)	0.8	0.017
HbA1c, % ^c^	5.4 (0.3)	5.5 (0.4)	5.6 (0.4)	3.7	<0.001
FBG, mg/dl ^c^	91.7 (8.6)	98.2 (9.8)	97.7 (9.6)	−5.0	<0.001

^a^ Standardized difference is the mean difference divided by the pooled standard deviation, expressed as a percentage.

^b^ Standardized differences were calculated using log-transformed triglyceride.

^c^ FBG or HbA1c or both can be measured in the program.

WC, waist circumference; BMI, body mass index; SBP, systolic blood pressure; DBP, diastolic blood pressure; TG, triglyceride; HDL, high-density lipoprotein cholesterol; HbA1c, hemoglobin A1c; and FBG, fasting blood glucose.

### Effectiveness of nationwide lifestyle intervention

**Fig 2:**
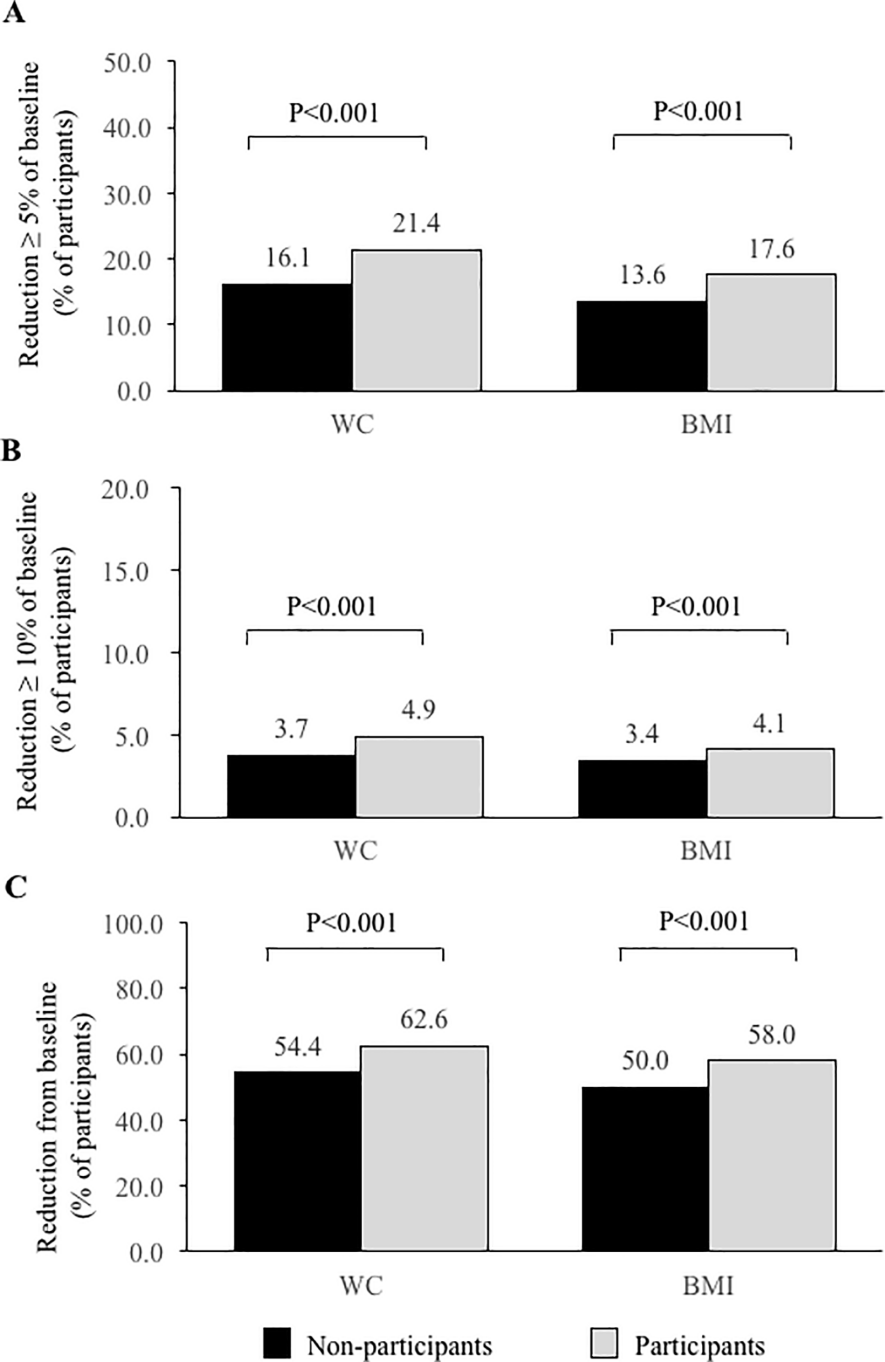
Categorical waist and weight reduction. WC, waist circumference; BMI, body mass index.

Clinically relevant reductions, i.e., ≥5% at year 3, were achieved in a significantly higher percentage of participants compared to non-participants (WC, 21.4% vs 16.1% and BMI, 17.6% vs 13.6%; p<0.001, each; **Fig 2**). Advanced weight reductions (≥10%) were also observed in a significantly higher percentage of participants. Both abdominal and overall obesity, measured by categorical weight reductions, improved significantly in participants compared to non-participants. Participants who received lifestyle intervention had significantly improvements in MetS, as compared with non-participants (reversal of MetS: 47.0% vs. 41.5%, p<0.001). Analysis in the matched cohort showed similar results (**S1 Fig**).

After we adjusted for confounders, lifestyle intervention was associated with an adjusted odds ratio (OR) 1.33 (95% confidence interval [CI]: 1.31–1.36, p<0.001) for 5% of WC reduction, 1.36 (95% CI 1.33–1.38, p<0.001) for 5% of BMI reduction, and 1.33 (95% CI: 1.31–1.33, p<0.001) for reversal of MetS (**Table 3**). Confirmatory analyses in a propensity-matched cohort yielded no substantive differences relative to traditional multivariate analyses (**Table 3**).

**Table 3:** Clinical end point in a whole cohort and a propensity-matched cohort.

	Unadjusted		Adjusted ^a^		Propensity-matched cohort	
	Odds ratios (95% CI)	P value	Odds ratios (95% CI)	P value	Odds ratios (95% CI)	P value
**Clinically relevant reduction (5%) in**						
**WC**	1.42 (1.39–1.44)	<0.001	1.33 (1.31–1.36)	<0.001	1.36 (1.33–1.40)	<0.001
**BMI**	1.36 (1.34–1.39)	<0.001	1.36 (1.33–1.38)	<0.001	1.38 (1.34–1.42)	<0.001
**Significant reduction (10%) in**						
**WC**	1.33 (1.29–1.37)	<0.001	1.24 (1.20–1.27)	<0.001	1.28 (1.21–1.34)	<0.001
**BMI**	1.23 (1.20–1.27)	<0.001	1.28 (1.24–1.32)	<0.001	1.34 (1.27–1.42)	<0.001
**Reduction in**						
**WC**	1.40 (1.38–1.42)	<0.001	1.33 (1.32–1.35)	<0.001	1.33 (1.31–1.36)	<0.001
**BMI**	1.38 (1.36–1.40)	<0.001	1.32 (1.30–1.33)	<0.001	1.31 (1.29–1.34)	<0.001
**Reversal of MetS**						
**Reversal of MetS**	1.33 (1.32–1.35)	<0.001	1.31 (1.29–1.33)	<0.001	1.27 (1.24–1.30)	<0.001

^a^ The control (non-participants) group is referent. WC; adjusted for age, sex, smoke, and waist circumferences at baseline. BMI; adjusted for age, sex, smoke, and body mass index at baseline. Reversal of MetS; adjusted for age, sex, body mass index, smoke, systolic blood pressure, log triglycerides, HDL-cholesterol, and HbA1c.

WC, waist circumference; BMI, body mass index; MetS, metabolic syndrome; CI, confidence interval.

**Fig 3:**
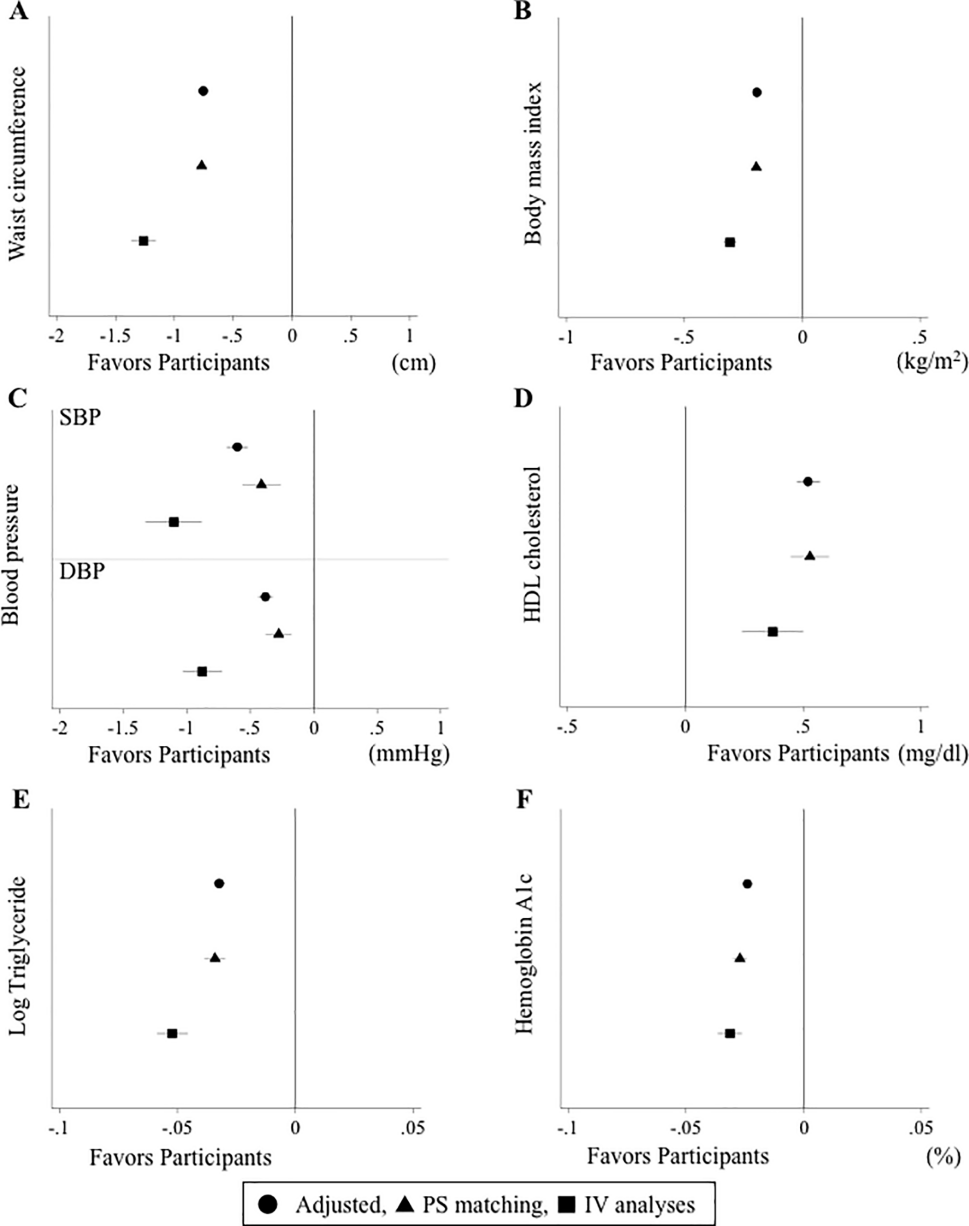
Beta-coefficients of each cardiometabolic risk factors using linear regression and instrumental variable methods. SBP, systolic blood pressure; DBP, diastolic blood pressure; PS, propensity score; IV, instrumental variable.

The mean WC changes were −1.34 and −0.44 cm in participants and non-participants, respectively, with a difference of −0.89 cm (95% CI: −0.92 to −0.86). The mean BMI changes were −0.29 and −0.08 kg/m^2^ in participants and non-participants, respectively, with a difference of −0.22 kg/m^2^ (95% CI: −0.22 to −0.21). The intervention program also resulted in significantly greater reductions in both abdominal and overall obesity parameters (**S1 Table**). Participants, compared to non-participants, had significant reductions in systolic blood pressure (SBP, −1.15 vs −0.72 mm Hg), diastolic blood pressure (DBP, −0.97 vs −0.64 mm Hg), and log TG (−0.11 vs −0.08), respectively (p<0.001 for each). Participants also improved their HDL-cholesterol level more than non-participants (1.48 vs 0.94 mg/dl). Of all parameters for obesity and cardiometabolic risk factors, greater reductions were observed in abdominal and overall obesity profiles, compared to cardiometabolic parameters. The adjusted differences in the traditional adjusted model were attenuated (ΔWC coefficient: −0.76; 95% CI: −0.79 to −0.73; ΔBMI coefficient: −0.19; −0.20 to −0.18), and the coefficients in the propensity-matched cohort were further attenuated (ΔWC: −0.77; −0.82 to −0.72; ΔBMI: −0.20; −0.21 to −0.18; **Fig 3**). The IV model strengthened the findings of the traditional adjusted model (**Fig 3**). Similar patterns were found for other cardiometabolic risk factors.

### Discussion

This study examined the effectiveness of a nationwide preventive program, which is comprised of screening, counselling, and education, using national “real world” data. Nationwide lifestyle intervention is effective in achieving clinically relevant reductions in WC and BMI and reversal of MetS. This intervention was also effective for long-term changes in cardiometabolic risk factors.

MetS is widely used to identify patients with abdominal obesity who have increased risk for CVD and diabetes [14]. While metabolic components likely overlap [15], we previously demonstrated that abdominal obesity is an independent predictor for new onset of individual MetS components in the longitudinal study [16]. In a previous study, abdominal obesity was found to be important in MetS development and preceded the development of other MetS components [17, 18]. Other researchers showed that abdominal obesity is superior to overall obesity for predicting total and cardiovascular mortality rates, suggesting that a reduction in abdominal obesity should reduce the risk of MetS and negative cardiovascular outcomes [19, 20].

A health promotion program focusing on lifestyles can substantially reduce obesity and CVD risk. However, the findings from previous reports have been equivocal. Despite the potentially large benefits of obesity reduction, lifestyle interventions have had small long-term success, especially at the population level [7, 21, 22]. In a previous meta-analysis, lifestyle interventions significantly reduced blood pressure and low-density lipoprotein cholesterol in the general population, but the pooled estimates have dubious validity because of marked unexplained heterogeneity among trials (*I*^*2*^ statistic >85%) [23, 24]. In the present study, we have shown long-term improvement in reducing obesity and cardiometabolic risk factors through lifestyle intervention within the framework of nationwide policy. These findings add further real-world evidence that suggests the nationwide preventive program that focuses on abdominal obesity was truly effective.

Although the randomized controlled study (RCT) unquestionably remains a powerful tool to minimize the risk of bias due to confounding factors and to develop scientific evidence, the usefulness of real-world evidence is emphasized through its potential for complementing the knowledge gained from clinical trials [25], where limitations make it difficult to generalize findings to larger nationwide settings [26]. In a previous population-based randomized lifestyle intervention study (the Inter99 study) [27], screening and lifestyle counselling had sustained effects on physical activity and dietary habits after the intervention discontinuation [28] and changes in physical activity level from baseline to 5-year follow-up were associated with relevant changes in weight, WC, DBP and serum lipids [29]. Nevertheless, there was no effect on incidence of diabetes [30] and development of fatal and non-fatal ischemic heart disease in the general population [31]. As the author discussed in the limitations, a 10-year follow-up could be too short to consider CVD. However, it is still early to conclude, based only on the randomized controlled trials [23, 31], if health checks followed by lifestyle counselling in a general population should not be part of a country’s health policy. Traditional RCTs are often conducted with specific populations and in a specialized environment that differ significantly from the realities of a public health policy setting and therefore RCTs might not provide a realistic view of policy impact for the general population [25]. Comprehensive and integrated public health prevention methods ideally attempt to implement multifaceted measures at each level of the health impact pyramid, which consists of five tiers: (1) socioeconomic factors; (2) changing the context to create healthy default decisions in the individual; (3) long-lasting protective interventions; (4) clinical interventions; and (5) counselling and education [32]. The Japanese national health policy is in line with the ideal of clinical care impacting population health. All Japanese people of the targeted age have equal opportunities to receive health checkups and lifestyle interventions under the law (tiers 1 and 5). The annual health checkup enables continuous monitoring (tier 3). Individuals at risk of developing CVD can receive counselling and educational interventions (tier 5), which can connect them to appropriate medical treatment (tier 2). The greatest differences between RCT and a public health policy setting is that the nationwide policy enables almost all citizens to learn the term “metabolic syndrome,” suggesting that it may be able to change the context of health by altering social norms (tiers 4 and 5). Moreover, this action has been conducted at the country level and led by the government, as recommended by the World Health Organization [33]. Therefore, the lifestyle intervention has maximized synergy, and we have achieved long-term success. In our analysis, we provide evidence that by implementing interventions, sustained public health and clinical benefits, as set forth at the beginning of the study, can be achieved. This study can lead to other successful population-based prevention strategies in a national policy context. Thus, the national lifestyle intervention can continue to yield a substantial and sustained reduction in abdominal obesity and cardiometabolic risk factors.

Our study had several limitations. Evaluating the effectiveness of the lifestyle interventions using non-randomized data has been controversial because of potential biases related to treatment selection (attending the lifestyle intervention or not) and the lack of data on potential confounding variables, including socioeconomic status, income, education, health literacy, or health motivation. The previous report suggested that there are two reasons for not attending the lifestyle interventions. Subjects may already have regular office visits, and subjects may not have time to attend the intervention. In the former scenario, non-participants must initiate lifestyle changes at their clinic, resulting in underestimated effectiveness of nationwide intervention. In the latter scenario, non-participants are not always less motivated than participants. Since participation increased more than doubled by shortening the intervention session time in the previous study, some non-participants may be willing to change their lifestyles on their own. Therefore, the latter reason may lead to either underestimation or overestimation of the intervention effect. To mitigate bias, we used three steps to assess intervention effectiveness. First, we controlled for demographic and clinical factors using a traditional multivariate model. Secondly, we used propensity-score matching to further control for selection bias. We applied all possible data believed to potentially affect the decision to attend lifestyle counselling in an effort to maximally reduce selection bias, which carefully balanced observed baseline characteristics [34]. If the distribution of unmeasured factors is more likely to be similar with similar clinical indications and risk when considering therapies, unmeasured factors can closely correlate with measured factors. In this setting, propensity scores may remove bias due to treatment selection differences resulting from unmeasured factors [10]. When strong selection bias exists, however, both multivariate adjustment and propensity-based matching are no more likely to remove bias due to unmeasured confounding variables. Thirdly, we have added IV analyses, which is a method designed to control for unmeasured bias. Our finding that several baseline characteristics modified the comparative effectiveness of lifestyle intervention is also consistent with the results of all analytical steps. Our analyses of nationwide observational data can provide reliable estimates of real-world lifestyle intervention effectiveness [35].

The second limitation includes the natural constraints of national data. Although a consistent format was used to collect data, the reported data were subject to data error and local variability. However, it was unlikely that the groups (i.e., participants and non-participants) would be differentially affected. Third, our subjects were selected based on attending checkups in both the first year and fiscal year 2011, indicating that they were health-conscious individuals. Our follow-up was 3 years, and the effects may attenuate over time. Fourth, we could not evaluate specific program features including individual vs. group sessions or physical activity vs. diet vs. both. Future research needs to identify which features optimize the effectiveness of the programs and which are less critical [36]. Fifth, the smoking status may be under-reported in self-answered questionnaires. Finally, the generalizability of our findings to other countries or ethnic groups with a higher burden of obesity and adverse cardiometabolic diseases is unknown. The aspects of Japanese culture may have contributed to our results, including the factors that already contribute to the very low obesity prevalence in Japan compared to other nations. It can also be difficult to adopt the current estimates worldwide due to a different MetS criteria in Japan. However, the United States Preventive Services Task Force recommends screening all adults for obesity and offering or referring obese adults to intensive behavioral interventions to improve weight status and other risk factors for important health outcomes [2]. In Finland, cardiovascular risk factor levels declined markedly after the implementation of a national CVD prevention strategy during the late 1970s [37]. Recently, the comprehensive healthy lifestyle program, comprising preventive and promotional activities that consider both population and high-risk approaches, has been applied to a middle-income country [38]. These studies introduce possible beneficial effects of nationwide lifestyle interventions in developed and developing countries. Further research is required to evaluate the long-term cost-effectiveness of a nationwide screening and lifestyle intervention. Clinical effectiveness and cost effectiveness underpinned by the best clinical evidence should be discussed in parallel [39].

In conclusion, the national screening and lifestyle intervention yielded a substantial and sustained improvement in abdominal obesity and reversal of MetS.

## Supporting information

S1 Appendix Create a database. (PDF)

S2 Appendix Methods for exams. (PDF)

S3 Appendix Information supply and lifestyle intervention. (PDF)

S4 Appendix Definition of urban and rural. (PDF)

S1 Fig Categorical Waist and Body Mass Index Loss for the matched cohort. (PDF)

S2 Fig Categorical Waist and Body Mass Index Loss by gender. (PDF)

S1 Table Total mean changes, standardized mean difference, and linear regression analyses of changes of metabolic syndrome components. (PDF)

S2 Table Selected baseline characteristics and outcomes across quartiles of facility lifestyle intervention ratio. (PDF)

S3 Table Baseline characteristics stratified by gender. (PDF)

S4 Table Clinical end point in a whole cohort and a propensity-matched cohort stratified by gender. (PDF)

S5 Table Total mean changes, standardized mean difference, and linear regression analyses of changes of metabolic syndrome components stratified by gender. (PDF)

## References

[1] Artinian NT, Fletcher GF, Mozaffarian D, Kris-Etherton P, Van Horn L, Lichtenstein AH, et al Interventions to promote physical activity and dietary lifestyle changes for cardiovascular risk factor reduction in adults: a scientific statement from the American Heart Association. Circulation. 2010;122(4):406–41. doi: 10.1161/CIR.0b013e3181e8edf1 . 20625115 10.1161/CIR.0b013e3181e8edf1 PMC6893884

[2] Moyer VA, Force USPST. Screening for and management of obesity in adults: U.S. Preventive Services Task Force recommendation statement. Annals of internal medicine. 2012;157(5):373–8. doi: 10.7326/0003-4819-157-5-201209040-00475 . 22733087 10.7326/0003-4819-157-5-201209040-00475

[3] Rothman AJ. Toward a theory-based analysis of behavioral maintenance. Health Psychol. 2000;19(1 Suppl):64–9. . 10709949 10.1037/0278-6133.19.suppl1.64

[4] Hu FB, Satija A, Manson JE. Curbing the Diabetes Pandemic: The Need for Global Policy Solutions. Jama. 2015;313(23):2319–20. doi: 10.1001/jama.2015.5287 . 25996138 10.1001/jama.2015.5287 PMC5291074

[5] Malik VS, Willett WC, Hu FB. Global obesity: trends, risk factors and policy implications. Nat Rev Endocrinol. 2013;9(1):13–27. doi: 10.1038/nrendo.2012.199 . 23165161 10.1038/nrendo.2012.199

[6] Bauer UE, Briss PA, Goodman RA, Bowman BA. Prevention of chronic disease in the 21st century: elimination of the leading preventable causes of premature death and disability in the USA. Lancet. 2014;384(9937):45–52. doi: 10.1016/S0140-6736(14)60648-6 . 24996589 10.1016/S0140-6736(14)60648-6

[7] Franz MJ, VanWormer JJ, Crain AL, Boucher JL, Histon T, Caplan W, et al Weight-loss outcomes: a systematic review and meta-analysis of weight-loss clinical trials with a minimum 1-year follow-up. J Am Diet Assoc. 2007;107(10):1755–67. doi: 10.1016/j.jada.2007.07.017 . 17904936 10.1016/j.jada.2007.07.017

[8] Alberti KG, Eckel RH, Grundy SM, Zimmet PZ, Cleeman JI, Donato KA, et al Harmonizing the metabolic syndrome: a joint interim statement of the International Diabetes Federation Task Force on Epidemiology and Prevention; National Heart, Lung, and Blood Institute; American Heart Association; World Heart Federation; International Atherosclerosis Society; and International Association for the Study of Obesity. Circulation. 2009;120(16):1640–5. doi: 10.1161/CIRCULATIONAHA.109.192644 . 19805654 10.1161/CIRCULATIONAHA.109.192644

[9] Wadden TA, Volger S, Sarwer DB, Vetter ML, Tsai AG, Berkowitz RI, et al A two-year randomized trial of obesity treatment in primary care practice. The New England journal of medicine. 2011;365(21):1969–79. doi: 10.1056/NEJMoa1109220 ; PubMed Central PMCID: PMCPMC3282598. 22082239 10.1056/NEJMoa1109220 PMC3282598

[10] Stukel TA, Fisher ES, Wennberg DE, Alter DA, Gottlieb DJ, Vermeulen MJ. Analysis of observational studies in the presence of treatment selection bias: effects of invasive cardiac management on AMI survival using propensity score and instrumental variable methods. Jama. 2007;297(3):278–85. doi: 10.1001/jama.297.3.278 ; PubMed Central PMCID: PMC2170524. 17227979 10.1001/jama.297.3.278 PMC2170524

[11] Garabedian LF, Chu P, Toh S, Zaslavsky AM, Soumerai SB. Potential bias of instrumental variable analyses for observational comparative effectiveness research. Annals of internal medicine. 2014;161(2):131–8. doi: 10.7326/M13-1887 . 25023252 10.7326/M13-1887

[12] Rassen JA, Mittleman MA, Glynn RJ, Alan Brookhart M, Schneeweiss S. Safety and effectiveness of bivalirudin in routine care of patients undergoing percutaneous coronary intervention. Eur Heart J. 2010;31(5):561–72. doi: 10.1093/eurheartj/ehp437 ; PubMed Central PMCID: PMCPMC2831765. 19942600 10.1093/eurheartj/ehp437 PMC2831765

[13] Normand ST, Landrum MB, Guadagnoli E, Ayanian JZ, Ryan TJ, Cleary PD, et al Validating recommendations for coronary angiography following acute myocardial infarction in the elderly: a matched analysis using propensity scores. Journal of clinical epidemiology. 2001;54(4):387–98. . 11297888 10.1016/s0895-4356(00)00321-8

[14] Eckel RH, Alberti KG, Grundy SM, Zimmet PZ. The metabolic syndrome. Lancet. 2010;375(9710):181–3. doi: 10.1016/S0140-6736(09)61794-3 . 20109902 10.1016/S0140-6736(09)61794-3

[15] Hwang LC, Bai CH, You SL, Sun CA, Chen CJ. Description and prediction of the development of metabolic syndrome: a longitudinal analysis using a markov model approach. PloS one. 2013;8(6):e67436 doi: 10.1371/journal.pone.0067436 ; PubMed Central PMCID: PMCPMC3688628. 23840701 10.1371/journal.pone.0067436 PMC3688628

[16] Nakao YM, Miyawaki T, Yasuno S, Nakao K, Tanaka S, Ida M, et al Intra-abdominal fat area is a predictor for new onset of individual components of metabolic syndrome: MEtabolic syndRome and abdominaL ObesiTy (MERLOT study). Proc Jpn Acad Ser B Phys Biol Sci. 2012;88(8):454–61. doi: 10.2183/pjab.88.454 ; PubMed Central PMCID: PMCPMC3491080. 23060233 10.2183/pjab.88.454 PMC3491080

[17] Cameron AJ, Boyko EJ, Sicree RA, Zimmet PZ, Soderberg S, Alberti KG, et al Central obesity as a precursor to the metabolic syndrome in the AusDiab study and Mauritius. Obesity (Silver Spring). 2008;16(12):2707–16. doi: 10.1038/oby.2008.412 . 18820650 10.1038/oby.2008.412

[18] Fox CS, Massaro JM, Hoffmann U, Pou KM, Maurovich-Horvat P, Liu CY, et al Abdominal visceral and subcutaneous adipose tissue compartments: association with metabolic risk factors in the Framingham Heart Study. Circulation. 2007;116(1):39–48. doi: 10.1161/CIRCULATIONAHA.106.675355 . 17576866 10.1161/CIRCULATIONAHA.106.675355

[19] Cerhan JR, Moore SC, Jacobs EJ, Kitahara CM, Rosenberg PS, Adami HO, et al A pooled analysis of waist circumference and mortality in 650,000 adults. Mayo Clin Proc. 2014;89(3):335–45. doi: 10.1016/j.mayocp.2013.11.011 ; PubMed Central PMCID: PMCPMC4104704. 24582192 10.1016/j.mayocp.2013.11.011 PMC4104704

[20] Sahakyan KR, Somers VK, Rodriguez-Escudero JP, Hodge DO, Carter RE, Sochor O, et al Normal-Weight Central Obesity: Implications for Total and Cardiovascular Mortality. Annals of internal medicine. 2015 doi: 10.7326/M14-2525 . 26551006 10.7326/M14-2525 PMC4995595

[21] Global Burden of Metabolic Risk Factors for Chronic Diseases C, Lu Y, Hajifathalian K, Ezzati M, Woodward M, Rimm EB, et al Metabolic mediators of the effects of body-mass index, overweight, and obesity on coronary heart disease and stroke: a pooled analysis of 97 prospective cohorts with 1.8 million participants. Lancet. 2014;383(9921):970–83. doi: 10.1016/S0140-6736(13)61836-X ; PubMed Central PMCID: PMCPMC3959199. 24269108 10.1016/S0140-6736(13)61836-X PMC3959199

[22] Douketis JD, Macie C, Thabane L, Williamson DF. Systematic review of long-term weight loss studies in obese adults: clinical significance and applicability to clinical practice. Int J Obes (Lond). 2005;29(10):1153–67. doi: 10.1038/sj.ijo.0802982 . 15997250 10.1038/sj.ijo.0802982

[23] Ebrahim S, Taylor F, Ward K, Beswick A, Burke M, Davey Smith G. Multiple risk factor interventions for primary prevention of coronary heart disease. Cochrane Database Syst Rev. 2011;(1):CD001561 doi: 10.1002/14651858.CD001561.pub3 . 21249647 10.1002/14651858.CD001561.pub3 PMC11729147

[24] Leblanc ES, O'Connor E, Whitlock EP, Patnode CD, Kapka T. Effectiveness of primary care-relevant treatments for obesity in adults: a systematic evidence review for the U.S. Preventive Services Task Force. Annals of internal medicine. 2011;155(7):434–47. doi: 10.7326/0003-4819-155-7-201110040-00006 . 21969342 10.7326/0003-4819-155-7-201110040-00006

[25] Sherman RE, Anderson SA, Dal Pan GJ, Gray GW, Gross T, Hunter NL, et al Real-World Evidence—What Is It and What Can It Tell Us? The New England journal of medicine. 2016;375(23):2293–7. Epub 2016/12/14. doi: 10.1056/NEJMsb1609216 . 27959688 10.1056/NEJMsb1609216

[26] Booth CM, Tannock IF. Randomised controlled trials and population-based observational research: partners in the evolution of medical evidence. Br J Cancer. 2014;110(3):551–5. Epub 2014/02/06. doi: 10.1038/bjc.2013.725 ; PubMed Central PMCID: PMCPMC3915111. 24495873 10.1038/bjc.2013.725 PMC3915111

[27] Jorgensen T, Borch-Johnsen K, Thomsen TF, Ibsen H, Glumer C, Pisinger C. A randomized non-pharmacological intervention study for prevention of ischaemic heart disease: baseline results Inter99. Eur J Cardiovasc Prev Rehabil. 2003;10(5):377–86. Epub 2003/12/10. doi: 10.1097/01.hjr.0000096541.30533.82 . 14663300 10.1097/01.hjr.0000096541.30533.82

[28] Baumann S, Toft U, Aadahl M, Jorgensen T, Pisinger C. The long-term effect of screening and lifestyle counseling on changes in physical activity and diet: the Inter99 Study—a randomized controlled trial. Int J Behav Nutr Phys Act. 2015;12:33 Epub 2015/04/18. doi: 10.1186/s12966-015-0195-3 ; PubMed Central PMCID: PMCPMC4352560. 25886540 10.1186/s12966-015-0195-3 PMC4352560

[29] Aadahl M, von Huth Smith L, Pisinger C, Toft UN, Glumer C, Borch-Johnsen K, et al Five-year change in physical activity is associated with changes in cardiovascular disease risk factors: the Inter99 study. Prev Med. 2009;48(4):326–31. Epub 2009/05/26. doi: 10.1016/j.ypmed.2009.01.015 . 19463487 10.1016/j.ypmed.2009.01.015

[30] Lau CJ, Pisinger C, Husemoen LLN, Jacobsen RK, Linneberg A, Jorgensen T, et al Effect of general health screening and lifestyle counselling on incidence of diabetes in general population: Inter99 randomised trial. Prev Med. 2016;91:172–9. Epub 2016/08/16. doi: 10.1016/j.ypmed.2016.08.016 . 27514243 10.1016/j.ypmed.2016.08.016

[31] Jorgensen T, Jacobsen RK, Toft U, Aadahl M, Glumer C, Pisinger C. Effect of screening and lifestyle counselling on incidence of ischaemic heart disease in general population: Inter99 randomised trial. Bmj. 2014;348:g3617 Epub 2014/06/11. doi: 10.1136/bmj.g3617 ; PubMed Central PMCID: PMCPMC4049194. 24912589 10.1136/bmj.g3617 PMC4049194

[32] Frieden TR. SHATTUCK LECTURE: The Future of Public Health. The New England journal of medicine. 2015;373(18):1748–54. doi: 10.1056/NEJMsa1511248 . 26510022 10.1056/NEJMsa1511248

[33] Organization WH. Noncommunicable diseases (NCD) [cited 2015 November 11]. Available from: http://www.who.int/gho/ncd/en/.

[34] Rosenbaum PR, Rubin DB. Reducing bias in observational studies using subclassification on the propensity score. J Am Stat Assoc. 1984;79:516–24.

[35] Hlatky MA, Boothroyd DB, Baker L, Kazi DS, Solomon MD, Chang TI, et al Comparative effectiveness of multivessel coronary bypass surgery and multivessel percutaneous coronary intervention: a cohort study. Annals of internal medicine. 2013;158(10):727–34. doi: 10.7326/0003-4819-158-10-201305210-00639 ; PubMed Central PMCID: PMCPMC4117804. 23609014 10.7326/0003-4819-158-10-201305210-00639 PMC4117804

[36] Balk EM, Earley A, Raman G, Avendano EA, Pittas AG, Remington PL. Combined Diet and Physical Activity Promotion Programs to Prevent Type 2 Diabetes Among Persons at Increased Risk: A Systematic Review for the Community Preventive Services Task Force. Annals of internal medicine. 2015;163(6):437–51. doi: 10.7326/M15-0452 ; PubMed Central PMCID: PMCPMC4692590. 26167912 10.7326/M15-0452 PMC4692590

[37] Vartiainen E, Jousilahti P, Alfthan G, Sundvall J, Pietinen P, Puska P. Cardiovascular risk factor changes in Finland, 1972–1997. Int J Epidemiol. 2000;29(1):49–56. . 10750603 10.1093/ije/29.1.49

[38] Sarrafzadegan N, Kelishadi R, Sadri G, Malekafzali H, Pourmoghaddas M, Heidari K, et al Outcomes of a comprehensive healthy lifestyle program on cardiometabolic risk factors in a developing country: the Isfahan Healthy Heart Program. Arch Iran Med. 2013;16(1):4–11. 013161/AIM.004. doi: 013161/AIM.004 . 23273227

[39] National Institute for Health and Care Excellence. Process and methods guides. The guidelines manual 2012 [cited 2017 December 15]. Available from: https://www.nice.org.uk/guidance/pmg6/resources/the-guidelines-manual-pdf-2007970804933.

