# Supplementary material for: Effectiveness of nationwide screening and lifestyle intervention for abdominal obesity and cardiometabolic risks in Japan: The metabolic syndrome and comprehensive lifestyle intervention study on nationwide database in Japan (MetS ACTION-J study)
Source: PLoS One. 2018 Jan 9;13(1):e0190862. doi: 10.1371/journal.pone.0190862 (PMC5760033; doi:10.1371/journal.pone.0190862)
Supplement: S1 Appendix — (PDF) [file pone.0190862.s001.pdf]

Supplementary Methods
Appendix S1: Create a database

The Ministry of Health, Labour, and Welfare (MHLW) in Japan has all data of participants of the 'tokuteikenshin' program in the National Database of Health Insurance Claims and Specific Health Checkups of Japan (NDB), which contains individual participant information. For anonymization, 2 unique identifications (IDs) have been included in the national database; the first ID (#1) is generated by health insurance code, birthdate, and gender, and the second ID (#2) is generated by name, birthdate, and gender.

In the present study, the MHLW provided 3-years (April, 2008–March, 2012) data of "tokutei" and "hoken" in CSV format. Datasets in each year included 3 different anonymous IDs: "tokutei" ID, "hoken" ID, and "tougou" ID (#1 and #2). "Tokutei" and "hoken" IDs are assigned randomly, whereas "tougo" ID (#1 and #2) is an unified ID, which is calculated by hash function using individual receipt information. Furthermore, this "tougou" ID is used to merge the "tokutei" and "hoken" datasets and combine datasets of different years.

In addition, the government classifies datasets into 4 parts: (1) basic information variables (i.e., age and gender), (2) basic medical checkup variables in "tokutei," (3) details of medical checkup variables in "tokutei," and (4) medical checkup variables after first medical check-up in "hoken." At first, the original datasets consist of aligning all variables vertically in each ID. Furthermore, continuous and categorical variables are included in different columns. Therefore, in order to modify information from one ID horizontally in a row, the columns in both continuous and categorical variables need to be transposed separately by each ID, and then merged back together. It is worth checking the type of content in all variables in each dataset because some continuous variables are defined to convert as categorical variables and vice versa.

We set outliers of greater or less than the mean ± 4 standard deviations as missing values because 99.7% of the data should be within 3 standard deviations of the mean. Furthermore, we excluded duplicative IDs in each year.

All statistical analyses were performed using SAS 9 (SAS Institute Inc., Cary, USA) and STATA 13.1 (College Station, TX, USA).

**Year**	**2008**	**2009**	**2010**	**2011**
Target population	51 919 920	52 211 735	52 192 070	52 534 157
Subjects who receive checkups	20 258 599	21 609 787	22 559 404	23 615 922
Our database	19 970 124	21 317 198	22 146 259	23 164 894
