# Supplementary material for: Effectiveness of nationwide screening and lifestyle intervention for abdominal obesity and cardiometabolic risks in Japan: The metabolic syndrome and comprehensive lifestyle intervention study on nationwide database in Japan (MetS ACTION-J study)
Source: PLoS One. 2018 Jan 9;13(1):e0190862. doi: 10.1371/journal.pone.0190862 (PMC5760033; doi:10.1371/journal.pone.0190862)
Supplement: S2 Appendix — (PDF) [file pone.0190862.s002.pdf]

Appendix S2: Methods for exams

All exams were conducted under Article prescribed by an Ordinance of the Ministry of Health, Labour and Welfare. (http://www.mhlw.go.jp/bunya/shakaihosho/iryouseido01/info02a.html) (Accessed March 28, 2017. [Japanese]).

Waist circumference: Refer to linked URLs (video) for details (http://www0.nih.go.jp/eiken/info/kokucho.html) (Accessed March 28, 2017. [Japanese]).

Blood pressure of the seated participant was measured twice after at least 5 min rest.
