# Supplementary material for: Effectiveness of nationwide screening and lifestyle intervention for abdominal obesity and cardiometabolic risks in Japan: The metabolic syndrome and comprehensive lifestyle intervention study on nationwide database in Japan (MetS ACTION-J study)
Source: PLoS One. 2018 Jan 9;13(1):e0190862. doi: 10.1371/journal.pone.0190862 (PMC5760033; doi:10.1371/journal.pone.0190862)
Supplement: S3 Appendix — (PDF) [file pone.0190862.s003.pdf]

Appendix S3: Information supply and lifestyle intervention

Information supply

Regardless of the presence/absence of the need of lifestyle intervention, information supply is ensured for ALL subjects of the specific health checkup once a year (simultaneously with notification of the checkup results) or more frequently. It provides information tailored to individual subjects based on the health checkup data and responses to the questionnaire filled in at the time of the checkup.

Major forms/means of support include the following:

- Individual explanation using a leaflet for information supply
- Enclosing the leaflet for information supply in the same envelope as that containing the checkup data
- Utilization of individual information supply pages in cases where Information and Communications Technology (ICT) is routinely used at the workplace
- Delivery of the leaflet for information supply at the time of the checkup data explanation meeting

Lifestyle intervention (positive support)

Support is provided in such a manner as to help the individual become aware of the lifestyle factors that require improvement, of the factors which should be further promoted, etc., and guiding the individual to take actions towards achieving the goal. The intervention is for 20 minutes or more when provided to each individual separately and for 80 minutes or more when provided to groups (8 individuals or less/group).

On the basis of the specific health checkup data and the results of the survey carried out to determine the status of lifestyle (smoking, exercise, diet, rest, etc), support is provided by interview and the results (evaluation made 6 months after the date of preparation of the action plan) are evaluated.

Lifestyle intervention (Intensive support)

At the beginning, support is provided via interview, followed by continuous support for 3 months or longer. Each session is for 20 minutes or more when provided to each individual separately and for 80 minutes or more when provided to groups (8 individuals or less/group).

On the basis of the specific health checkup data and results of the survey carried out to determine the lifestyle status (smoking, exercise, diet, rest, etc.), support by interview is provided, and the progress of the action plan implementation (interim evaluation) and the results (6 months after the date of preparation of the action plan) are evaluated.

Table. Stratification for lifestyle intervention by risks.

**Abdominal obesity**	**Risks**<br>**1. High BP**<br>**2. Dyslipidemia**<br>**3. High FBG**	**Smoking**	**Age: 40 – 64 years**	**Age: 65 – 74 years**
No abdominal obesity and BMI <25 kg/m^2	Any risk		Information supply only	
≥ 85 cm (men)<br>≥ 90 cm (women)	≥ 2 risks		Information supply & Intervention (Positive support)	Information supply & Intervention (Intensive support)
	1 risk	Yes		
		No		
No abdominal obesity, but BMI ≥25 kg/m^2	3 risks		Information supply & Intervention (Positive support)	Information supply & Intervention (Intensive support)
	2 risks	Yes		
		No		
	1 risk			

BP, blood pressure; FBG, fasting blood glucose; BMI, body mass index.
