# Supplementary material for: Effectiveness of nationwide screening and lifestyle intervention for abdominal obesity and cardiometabolic risks in Japan: The metabolic syndrome and comprehensive lifestyle intervention study on nationwide database in Japan (MetS ACTION-J study)
Source: PLoS One. 2018 Jan 9;13(1):e0190862. doi: 10.1371/journal.pone.0190862 (PMC5760033; doi:10.1371/journal.pone.0190862)

## Appendix S4: Definition of urban and rural

Information regarding the location of residence was only available at the prefecture level; therefore, we defined urban and rural based on Densely Inhabited Districts (DID). DID are the urban areas that are designated based on statistical data and meet the following criteria:

- A district containing basic unit blocks, etc. with a population density of ≥4,000 per square kilometer; such districts are adjacent to each other in a municipality.
- A district consisting of the above adjacent basic unit blocks, etc. with a population of ≥5,000 at the time of the Population Census of Japan.

(http://www.stat.go.jp/english/data/chiri/did/1-1.htm)

In 2010, the most recent year available, the national average percentage of the DID population was 67.3% according the Statistics Japan. We used the national average as the basis for urban areas.

Urban: Proportion of the DID population was higher than the national average (67.3%) in 2010 (*blue* area on a map). Tokyo (98.2%), Osaka (95.8%), Kanagawa (94.2%), Kyoto (83.0%), Saitama (79.6%), Aichi (76.8%), Hyogo (76.6%), Hokkaido (74.0%), Chiba (72.9%), and Fukuoka (70.9%) were considered urban areas.

Rural: Proportion of the DID population was lower than the national average in 2010 (*white* area on a map).

This map was created by MANDARA ver. 9.40 (http://ktgis.net/mandara/).

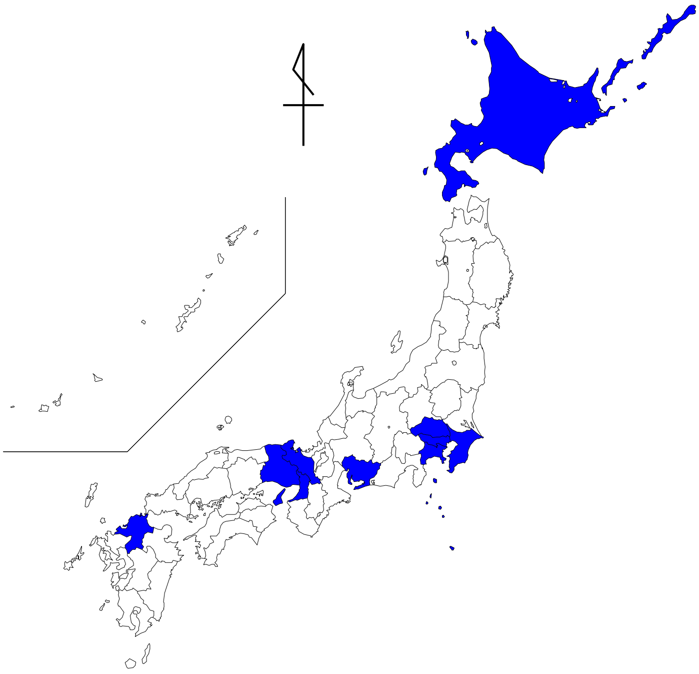

Supplement: S4 Appendix — (PDF) [file pone.0190862.s004.pdf]
