# Supplementary material for: Effectiveness of nationwide screening and lifestyle intervention for abdominal obesity and cardiometabolic risks in Japan: The metabolic syndrome and comprehensive lifestyle intervention study on nationwide database in Japan (MetS ACTION-J study)
Source: PLoS One. 2018 Jan 9;13(1):e0190862. doi: 10.1371/journal.pone.0190862 (PMC5760033; doi:10.1371/journal.pone.0190862)
Supplement: S2 Fig — (PDF) [file pone.0190862.s006.pdf]

Figure S2. Categorical Waist and Body Mass Index Loss by gender

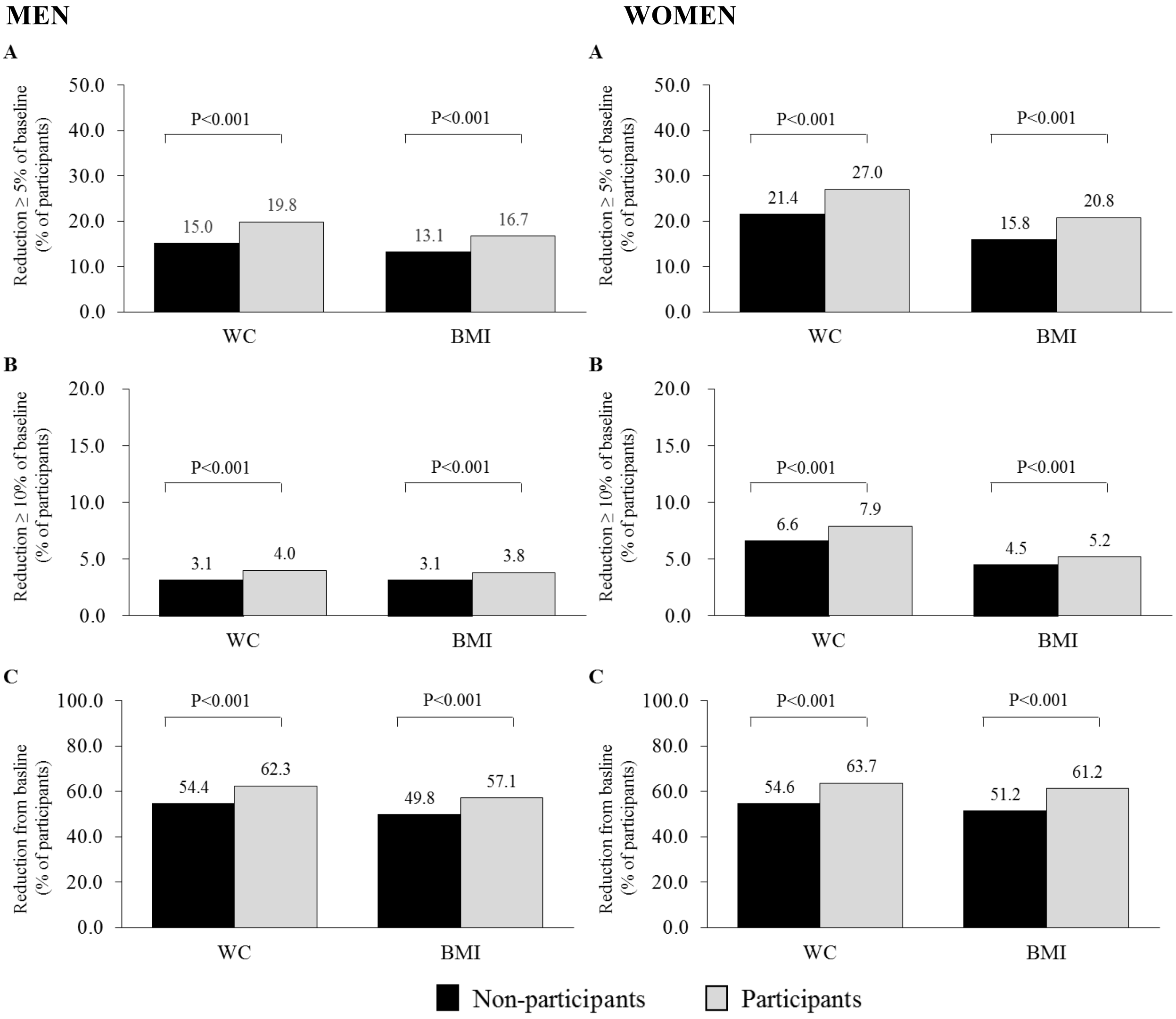

Panel A shows the percentage of participants who lost 5% or more of their baseline WC (waist circumferences) or BMI (body mass index), Panel B shows the percentage of participants who lost 10% or more of their baseline WC or BMI, and Panel C shows the percentage of participants in each group who were at or below their baseline WC (waist circumference) or BMI (body mass index) after 3 years.
