# Supplementary material for: Effectiveness of nationwide screening and lifestyle intervention for abdominal obesity and cardiometabolic risks in Japan: The metabolic syndrome and comprehensive lifestyle intervention study on nationwide database in Japan (MetS ACTION-J study)
Source: PLoS One. 2018 Jan 9;13(1):e0190862. doi: 10.1371/journal.pone.0190862 (PMC5760033; doi:10.1371/journal.pone.0190862)
Supplement: S1 Table — (PDF) [file pone.0190862.s007.pdf]

Table S1. Total mean changes, standardized mean difference, and linear regression analyses of changes of metabolic syndrome components

Characteristics [a]	Non-participants	Participants	Differences (95% CI) [b]	Crude β by 1 SD increase (95% CI) †	Multivariate adjusted β by 1 SD increase (95% CI) ‡
ΔWC, cm	-0.44	-1.34	-0.89 (-0.92 to -0.86)	-0.19 (-0.19 to -0.18)	-0.16 (-0.17 to -0.15)
ΔBMI, kg/m^2	-0.08	-0.29	-0.22 (-0.22 to -0.21)	-0.16 (-0.17 to -0.16)	-0.14 (-0.15 to -0.14)
ΔSBP, mm Hg	-0.72	-1.15	-0.43 (-0.53 to -0.34)	-0.03 (-0.04 to -0.02)	-0.04 (-0.05 to -0.03)
ΔDBP, mm Hg	-0.64	-0.97	-0.33 (-0.39 to -0.26)	-0.03 (-0.04 to -0.02)	-0.04 (-0.04 to -0.03)
ΔlnTG, mg/dl	-0.08	-0.11	-0.03 (-0.04 to -0.03)	-0.08 (-0.08 to -0.07)	-0.07 (-0.08 to -0.07)
ΔHDL, mg/dl	0.94	1.48	0.54 (0.49 to 0.59)	0.07 (0.06 to 0.07)	0.06 (0.06 to 0.07)
ΔFBG, mg/dl	0.90	0.14	-0.76 (-0.84 to -0.68)	-0.08 (-0.08 to -0.07)	-0.08 (-0.08 to -0.07)
ΔHbA1c, %	0.07	0.04	-0.03 (-0.03 to -0.03)	-0.10 (-0.10 to -0.09)	-0.08 (-0.09 to -0.07)

[a] Delta gap denotes variables in 2011 minus variables in 2008.

[b] The control (non-participants) group is referent. One standard deviation is 4.7472 for waist circumference, 1.3220 for body mass index, 15.0317 for systolic blood pressure, 10.5321 for diastolic blood pressure, 0.4353 for log triglyceride, 8.1036 for HDL cholesterol, 10.0365 for fasting blood glucose, and 0.2954 for HbA1c.

CI, confidence interval; SD, standard deviation; WC, waist circumference; BMI, body mass index; SBP, systolic blood pressure; DBP, diastolic blood pressure; TG, triglyceride; HDL, high-density lipoprotein cholesterol; FBG, fasting blood glucose.
