# Supplementary material for: Effectiveness of nationwide screening and lifestyle intervention for abdominal obesity and cardiometabolic risks in Japan: The metabolic syndrome and comprehensive lifestyle intervention study on nationwide database in Japan (MetS ACTION-J study)
Source: PLoS One. 2018 Jan 9;13(1):e0190862. doi: 10.1371/journal.pone.0190862 (PMC5760033; doi:10.1371/journal.pone.0190862)
Supplement: S2 Table — (PDF) [file pone.0190862.s008.pdf]

Table S2. Selected baseline characteristics and outcomes across quartiles of facility lifestyle intervention ratio

	Quartile (range) of faculty lifestyle intervention ratio, %[a]			
	Q1<br>(0.1 - 4.8)	Q2<br>(4.8 - 7.9)	Q3<br>(7.9 - 15.8)	Q4<br>(15.8 - 60.0)
n	220 048	222 172	217 928	220 006
Age, %				
40-45	26.6	27.2	26.4	18.9
45-50	26.5	26.4	24.7	19.0
50-55	24.1	22.9	21.3	17.9
55-60	14.6	14.1	13.4	13.5
60-65	4.5	5.0	6.2	10.6
65-70	2.9	3.4	6.0	14.8
>70	0.8	1.0	2.0	5.3
Gender, %	85.5	86.7	83.2	76.1
Smoking, %	36.9	36.8	35.3	29.6
Mean				
WC, cm	91.3	91.2	91.2	91.3
BMI, kg/m^2	26.2	26.2	26.2	26.1
SBP, mm Hg	129.8	130.3	130.4	131.5
DBP, mm Hg	82.2	82.7	82.2	81.4
TG, mg/dl [b]	147	150	148	142
HDL, mg/dl	54.6	54.1	54.0	54.2
FBG, mg/dl	97.7	98.0	98.2	97.3
HbA1c, %	5.5	5.6	5.5	5.6
Prevalence of 5% reduction in (95%CI), %				
WC	15.4 (15.3 - 15.6)	15.8 (15.6 - 15.9)	16.8 (16.6 - 16.9)	18.3 (18.1 - 18.4)
BMI	13.2 (13.1 - 13.4)	13.4 (13.3 - 13.6)	14.2 (14.1 - 14.3)	15.1 (14.9 - 15.2)
Adjusted odds ratio (95% CI)				
Reversal of MetS	Referent	1.01 (0.99 - 1.02)	1.05 (1.04 - 1.07)	1.17 (1.15 - 1.18)
β coefficients				
⊿WC	Referent	-0.03	-0.17	-0.30
⊿BMI	Referent	-0.01	-0.04	-0.08

[a] Although there were small differences in age distribution, cardiometabolic characteristics was remarkably similar across regions. The balance in the distribution of all measured risk factors across regions provides

reasonable evidence to infer that the distribution of unmeasured risk factors is likely balanced across regions as well (Stukel TA, et al. JAMA. 2007;297(3):278-85.). The wide range of lifestyle intervention rates (0.1 - 60%) and the similarity in cardiometabolic characteristics lend support to faculty lifestyle intervention rates being a valid instrumental variable.

[b] Median.

WC, waist circumference; BMI, body mass index; SBP, systolic blood pressure; DBP, diastolic blood pressure; TG, triglyceride; HDL, high-density lipoprotein cholesterol; HbA1c, haemoglobin A1c; and FBG, fasting blood glucose; CI, confidence interval.
