# Supplementary material for: Effectiveness of nationwide screening and lifestyle intervention for abdominal obesity and cardiometabolic risks in Japan: The metabolic syndrome and comprehensive lifestyle intervention study on nationwide database in Japan (MetS ACTION-J study)
Source: PLoS One. 2018 Jan 9;13(1):e0190862. doi: 10.1371/journal.pone.0190862 (PMC5760033; doi:10.1371/journal.pone.0190862)
Supplement: S3 Table — (PDF) [file pone.0190862.s009.pdf]

Table S3. Baseline characteristics stratified by gender

Characteristics	Overall cohort			Propensity-score matched cohort	
	No risk group (eligible for healthcare guidance)	Risk group (eligible for interventional program)		Risk group (eligible for interventional program)	
		Non-participants	Participants	Non-participants	Participants
**Men**					
n	1 726 967	747 963	86 969	59 553	59 566
Age, %					
40-45	30.6	27.0	22.3	22.3	22.8
45-50	25.0	25.5	22.9	22.6	23.6
50-55	19.6	22.1	19.9	20.0	20.7
55-60	11.8	13.6	13.1	13.7	13.7
60-65	5.2	5.3	7.1	7.8	6.7
65-70	5.8	4.9	10.7	10.0	9.1
>70	1.9	1.7	4.1	3.5	3.4
Smoking	39.6	40.4	35.1	36.1	36.3
WC, cm	79.7 (6.1)	91.1 (5.7)	90.9 (5.5)	90.9 (5.4)	90.8 (5.3)
BMI, kg/m^2	22.1 (2.2)	26.0 (2.4)	25.9 (2.3)	25.9 (2.4)	25.9 (2.3)
SBP, mm Hg	120.0 (14.9)	130.1 (15.7)	129.8 (15.1)	130.0 (15.7)	129.8 (15.1)
DBP, mm Hg	75.2 (10.4)	82.5 (11.0)	81.7 (10.5)	81.7 (10.8)	81.7 (10.5)
TG, mg/dl	94 (68 - 129)	153 (105 - 208)	151 (102 - 204)	153 (105 - 208)	151 (102 - 204)
HDL, mg/dl	61.7 (15.0)	52.7 (12.6)	52.6 (12.5)	52.5 (12.7)	52.6 (12.6)
FBG, mg/dl	93.3 (8.7)	98.5 (9.8)	98.0 (9.6)	98.7 (9.8)	98.2 (9.6)
HbA1c, %	5.4 (0.3)	5.5 (0.4)	5.5 (0.4)	5.5 (0.4)	5.5 (0.4)
**Women**					
n	1 623 387	159 946	24 810	16 224	16 211
Age, %					
40-45	23.9	15.1	7.0	10.0	7.8
45-50	21.5	17.9	9.1	13.9	10.0
50-55	18.1	19.8	11.6	15.1	13.0
55-60	12.6	15.6	13.6	12.9	15.1
60-65	10.2	12.0	21.0	15.4	20.9
65-70	10.6	14.5	28.1	24.0	25.0

>70	3.1	5.0	9.7	8.7	8.2
Smoking	9.4	9.5	4.8	7.3	5.2
WC, cm	76.5 (7.6)	91.9 (7.0)	92.3 (6.5)	92.0 (6.7)	92.1 (6.5)
BMI, kg/m^2	21.1 (2.4)	27.1 (2.8)	26.7 (2.6)	26.8 (2.7)	26.7 (2.6)
SBP, mm Hg	116.4 (16.2)	133.3 (16.9)	133.4 (16.1)	133.2 (16.7)	133.5 (16.3)
DBP, mm Hg	70.8 (10.6)	80.7 (11.0)	79.4 (10.3)	79.7 (10.9)	79.7 (10.4)
TG, mg/dl	72 (54 - 99)	114 (81 - 164)	115 (83 - 163)	114 (81 - 164)	115 (83 - 163)
HDL, mg/dl	71.2 (15.6)	60.3 (13.8)	59.5 (13.5)	59.9 (13.7)	59.7 (13.6)
FBG, mg/dl	90.0 (8.1)	97.0 (9.7)	96.7 (9.5)	96.9 (9.8)	96.6 (9.6)
HbA1c, %	5.4 (0.3)	5.6 (0.3)	5.6 (0.3)	5.6 (0.3)	5.6 (0.3)

WC, waist circumference; BMI, body mass index; SBP, systolic blood pressure; DBP, diastolic blood pressure; TG, triglyceride; HDL, high-density lipoprotein cholesterol; HbA1c, haemoglobin A1c; and FBG, fasting blood glucose.
