# Supplementary material for: Effectiveness of nationwide screening and lifestyle intervention for abdominal obesity and cardiometabolic risks in Japan: The metabolic syndrome and comprehensive lifestyle intervention study on nationwide database in Japan (MetS ACTION-J study)
Source: PLoS One. 2018 Jan 9;13(1):e0190862. doi: 10.1371/journal.pone.0190862 (PMC5760033; doi:10.1371/journal.pone.0190862)
Supplement: S4 Table — (PDF) [file pone.0190862.s010.pdf]

Table S4. **Clinical end point in a whole cohort and a propensity-matched cohort stratified by gender**

	**Unadjusted**		**Adjusted** [a]		**Propensity-matched cohort**	
	**Odds ratios (95% CI)**	**P value**	**Odds ratios (95% CI)**	**P value**	**Odds ratios (95% CI)**	**P value**
**Men**						
Clinically relevant reduction (5%) in						
WC	1.40 (1.37 - 1.42)	<0.001	1.38 (1.35 - 1.40)	<0.001	1.43 (1.38 - 1.48)	<0.001
BMI	1.33 (1.31 - 1.36)	<0.001	1.35 (1.32 - 1.37)	<0.001	1.37 (1.33 - 1.42)	<0.001
Significant reduction (10%) in						
WC	1.31 (1.26 - 1.35)	<0.001	1.32 (1.28 - 1.37)	<0.001	1.35 (1.27 - 1.45)	<0.001
BMI	1.23 (1.18 - 1.28)	<0.001	1.28 (1.24 - 1.33)	<0.001	1.35 (1.26 - 1.45)	<0.001
Reduction in						
WC	1.38 (1.36 - 1.40)	<0.001	1.34 (1.32 - 1.36)	<0.001	1.37 (1.33 - 1.40)	<0.001
BMI	1.34 (1.32 - 1.36)	<0.001	1.30 (1.29 - 1.32)	<0.001	1.31 (1.28 - 1.34)	<0.001
Reversal of MetS	1.34 (1.32 - 1.37)	<0.001	1.34 (1.31 - 1.36)	<0.001	1.29 (1.25 - 1.33)	<0.001
**Women**						
Clinically relevant reduction (5%) in						
WC	1.36 (1.32 - 1.40)	<0.001	1.17 (1.14 - 1.21)	<0.001	1.23 (1.14 - 1.34)	<0.001
BMI	1.40 (1.35 - 1.45)	<0.001	1.37 (1.32 - 1.41)	<0.001	1.45 (1.32 - 1.59)	<0.001
Significant reduction (10%) in						
WC	1.23 (1.17 - 1.29)	<0.001	1.05 (1.00 - 1.11)	0.047	1.26 (1.11 - 1.44)	0.001
BMI	1.16 (1.10 - 1.24)	<0.001	1.25 (1.18 - 1.33)	<0.001	1.20 (1.01 - 1.42)	0.037
Reduction in						
WC	1.46 (1.42 - 1.51)	<0.001	1.27 (1.23 - 1.30)	<0.001	1.29 (1.20 - 1.39)	<0.001

BMI	1.51 (1.46 - 1.55)	<0.001	1.35 (1.31 - 1.39)	<0.001	1.37 (1.28 - 1.48)	<0.001
Reversal of MetS	1.24 (1.20 - 1.27)	<0.001	1.23 (1.19 - 1.27)	<0.001	1.22 (1.12 - 1.32)	<0.001

[a] The control (non-participants) group is referent. WC; adjusted for age, gender, smoke, and waist circumferences at baseline. BMI; adjusted for age, gender, smoke, and body mass index at baseline. Reversal of MetS; adjusted for age, gender, body mass index, smoke, systolic blood pressure, log triglycerides, HDL-cholesterol, and HbA1c.

WC, waist circumference; BMI, body mass index; MetS, metabolic syndrome; CI, confidence interval.
