# Supplementary material for: Effectiveness of nationwide screening and lifestyle intervention for abdominal obesity and cardiometabolic risks in Japan: The metabolic syndrome and comprehensive lifestyle intervention study on nationwide database in Japan (MetS ACTION-J study)
Source: PLoS One. 2018 Jan 9;13(1):e0190862. doi: 10.1371/journal.pone.0190862 (PMC5760033; doi:10.1371/journal.pone.0190862)
Supplement: S5 Table — (PDF) [file pone.0190862.s011.pdf]

Table S5. Total mean changes, standardized mean difference, and linear regression analyses of changes of metabolic syndrome components stratified by gender

Characteristics [a]	Non-participants	Participants	Differences (95% CI) [b]	Crude β by 1 SD increase (95% CI) †	Multivariate adjusted β by 1 SD increase (95% CI) ‡
**Men**					
ΔWC, cm	-0.42	-1.24	-0.82 (-0.85 to -0.79)	-0.18 (-0.19 to -0.18)	-0.17 (-0.18 to -0.16)
ΔBMI, kg/m^2	-0.07	-0.27	-0.20 (-0.21 to -0.19)	-0.15 (-0.16 to -0.15)	-0.14 (-0.15 to -0.14)
ΔSBP, mm Hg	-0.54	-0.91	-0.37 (-0.48 to -0.27)	-0.03 (-0.03 to -0.02)	-0.04 (-0.05 to -0.03)
ΔDBP, mm Hg	-0.45	-0.72	-0.27 (-0.35 to -0.20)	-0.03 (-0.03 to -0.02)	-0.04 (-0.04 to -0.03)
ΔlnTG, mg/dl	-0.08	-0.12	-0.03 (-0.04 to -0.03)	-0.08 (-0.09 to -0.07)	-0.08 (-0.09 to -0.07)
ΔHDL, mg/dl	0.97	1.54	0.58 (0.52 to 0.63)	0.07 (0.06 to 0.08)	0.07 (0.06 to 0.08)
ΔFBG, mg/dl	0.99	0.30	-0.69 (-0.78 to -0.60)	-0.07 (-0.08 to -0.06)	-0.07 (-0.08 to -0.07)
ΔHbA1c, %	0.07	0.05	-0.03 (-0.03 to -0.02)	-0.09 (-0.10 to -0.08)	-0.08 (-0.09 to -0.07)
**Women**					
ΔWC, cm	-0.53	-1.66	-1.13 (-1.21 to -1.05)	-0.19 (-0.20 to -0.18)	-0.11 (-0.12 to -0.09)
ΔBMI, kg/m^2	-0.12	-0.39	-0.28 (-0.30 to -0.26)	-0.18 (-0.20 to -0.17)	-0.14 (-0.15 to -0.13)
ΔSBP, mm Hg	-1.57	-2.00	-0.43 (-0.65 to -0.22)	-0.03 (-0.04 to -0.01)	-0.04 (-0.05 to -0.03)
ΔDBP, mm Hg	-1.51	-1.81	-0.31 (-0.45 to -0.16)	-0.03 (-0.04 to -0.02)	-0.03 (-0.02-4 to -0.02)
ΔlnTG, mg/dl	-0.05	-0.08	-0.04 (-0.04 to -0.03)	-0.09 (-0.11 to -0.08)	-0.06 (-0.07 to -0.05)
ΔHDL, mg/dl	0.81	1.27	0.46 (0.35 to 0.57)	0.05 (0.04 to 0.07)	0.04 (0.05 to 0.03)
ΔFBG, mg/dl	0.42	-0.52	-0.94 (-1.12 to -0.76)	-0.10 (-0.12 to -0.08)	-0.08 (-0.10 to -0.06)
ΔHbA1c, %	0.07	0.03	-0.03 (-0.04 to -0.03)	-0.12 (-0.13 to -0.10)	-0.07 (-0.08 to -0.05)

[a] Delta gap denotes variables in 2011 minus variables in 2008.

[b] The control (non-participants) group is referent. One standard deviation (SD) in men is 4.4464 for waist circumference, 1.2770 for body mass index, 14.7436 for systolic blood pressure, 10.4822 for diastolic blood pressure, 0.4398 for log triglyceride, 8.0190 for HDL cholesterol, 10.1095 for fasting blood glucose, and 0.2970 for HbA1c. One SD in women is 5.9150 for waist circumference, 1.5065 for body mass index, 16.2412 for systolic blood pressure, 10.7105 for diastolic blood pressure, 0.4137 for log triglyceride, 8.4736 for HDL cholesterol, 9.6442 for fasting blood glucose, and 0.2884 for HbA1c.

CI, confidence interval; SD, standard deviation; WC, waist circumference; BMI body mass index; SBP, systolic blood pressure; DBP, diastolic blood pressure; TG, triglyceride; HDL, high-density lipoprotein cholesterol; FBG, fasting blood glucose.
